# Cervical cancer prevention by vaccination: review

**DOI:** 10.3389/fonc.2024.1386167

**Published:** 2024-04-23

**Authors:** Julio Cesar González-Rodríguez, Aurelio Cruz-Valdez, Vicente Madrid-Marina

**Affiliations:** ^1^ Department of Oncology Gynecology, Instituto Nacional de Cancerología (INCAN), México City, Mexico; ^2^ Center for Population Health Research, Instituto Nacional de Salud Pública (INSP), Cuernavaca, Mexico; ^3^ Chronic Infections and Cancer Division, Center for Research on Infectious Diseases, Instituto Nacional de Salud Pública (INSP), Cuernavaca, Mexico

**Keywords:** human papillomaviruses, HPV, Prophylactic HPV vaccines, effectiveness, HPV Vaccines

## Abstract

**Abstract:**

Routine use of human papillomavirus (HPV) vaccines is recommended in adolescents under 15 years of age worldwide. Still, effective programs remain suboptimal for several factors, making the WHO strategy to eradicate cervical cancer public health with an uncertain future.

**Objective:**

To review the literature on the effectiveness, long-term protection, and safety of HPV vaccination programs and vaccination as adjuvant management. This review aims to describe the current state of vaccination programs and demonstrate the long-term protection and safety of vaccines implemented worldwide targeting adolescent girls, with the most recent published evidence of the three prophylactic HPV vaccines – bivalent (bHPV), quadrivalent (qHPV), and nonavalent (nHPV)-. We mainly focus on publications evaluating efficacy, dosing schemes, and HPV vaccination, as well as studies contributing to the mounting evidence for the real-life effectiveness of prophylactic HPV vaccines from several countries.

**Findings:**

Human Papillomavirus vaccination programs have made remarkable strides in preventing HPV-related diseases; countries with robust vaccination efforts have witnessed substantial reductions in HPV-related diseases with a decline in high-grade cervical abnormalities and genital warts (54%-83%). However, global coverage remains uneven, with disparities between high-income (HICs) and low-income countries (LMICs). The long-term efficacy of the available human papillomavirus (HPV) goes up to 9.4 years and continues to be immunogenic and well tolerated with an excellent safety profile.

**Conclusions and relevance:**

As these are crucial topics in HPV vaccination, it is essential to establish systems for continued monitoring of vaccine immunogenicity, efficacy, and safety over time.

## 1 Introduction

Cervical cancer (CC) poses a significant public health issue. To combat this problem, routine vaccination against human papillomavirus (HPV) is strongly encouraged globally for adolescents younger than 15. However, the effectiveness of these programs is often compromised by various factors.

To address this, a systematic literature review was conducted using the PubMed database, focusing on research articles published between January 2020 and April 2023. The search strategy involved combining the term “cervical cancer” with “prevention” and “vaccination.” Priority was given to controlled, randomized studies and systematic reviews with meta-analysis. However, all available evidence was considered when sufficient studies were unavailable.

### 1.1 Importance of HPV detection for cervical cancer prevention

Cervical cancer is the fourth most common cancer in women worldwide, with a projected 604,000 new cases and 342,000 deaths in 185 countries by 2020 (1). Furthermore, data obtained from the Mexican Burden of Disease Study (MBD-2013) demonstrated that there were 102,241 cases of cancer in women, with CC being the second most common type of cancer after breast cancer. The age-standardized incidence rate for CC was 12 per 100,000, resulting in 12,562 new cases. A ranking of age-standardized mortality rates among Mexican women showed that CC was the leading cause of death in states with higher levels of marginalization. These findings highlight the significant burden of CC in Mexico, particularly in marginalized communities, underscoring the need for targeted interventions to address this public health issue (2).

The global prevalence of HPV infection among women without cervical abnormalities ranges between 11 and 12%, with notable variations across regions. Sub-Saharan Africa (24%), Eastern Europe (21%), and Latin America (16%) exhibit the highest rates (3). Age-stratified HPV distribution patterns reveal a peak among younger individuals (<25 years) and a resurgence in older age groups (>45 years), specifically in the Americas and Africa. The most commonly detected HPV genotypes are HPV-16 (3.2%), HPV-18 (1.4%), HPV-52 (0.9%), HPV-31 (0.8%), and HPV-58 (0.7%) (4). The prevalence of oncogenic HPV genotypes is directly correlated with the severity of cervical lesions and the burden of HPV infection, highlighting the association between these genotypes and the progression to cancer (5). This link is further supported by the observation that cervical cancer is predominantly caused by the high-risk HPV genotypes, particularly HPV-16 and HPV-18 (6). A study conducted in Mexico on a population of 60,135 women found that 24.78% of them had high-risk human papillomavirus (HR HPV). Among the HR HPV types, the most prevalent were HPV-16 (4.13%), HPV-31 (4.12%), and HPV-51 (3.39%). On the other hand, HPV-18 (1.70%) was less common among the infected women (7, 8).

In Mexico, CC prevention and control are guided by the official standard NOM-014-SSA2-1994, endorsed by the national government. This standard encompasses prevention, early detection, diagnosis, treatment, control, and epidemiological surveillance of CC. The program for early CC detection includes a Papanicolaou smear test, complemented by biomolecular tests for human papillomavirus (HPV) detection as an auxiliary tool to cervical cytology. Acetic acid-based direct visualization is also employed when the Papanicolaou smear is unavailable. These screening tests are recommended for all asymptomatic women aged 25-64. However, women under 25 or over 64 years of age with decreased morbidity and specific risk factors associated with CC should also undergo testing (9).

The CC Action Program was initiated to decrease cervical cancer mortality rates in Mexican females. To achieve this objective, various strategic actions have been taken, encompassing collaboration, fostering inter-sectoral and intra-sectoral coordination to streamline efforts and resources; early detection, enhancing timely identification and screening for cervical cancer, ensuring prompt diagnosis and treatment; quality control, implementing rigorous quality control measures to provide accurate and reliable diagnosis and treatment; supervision and evaluation, establishing adequate supervision and evaluation mechanisms to monitor program implementation, assess outcomes, and drive continuous improvement; research and development, promoting research and innovation to advance understanding of cervical cancer, develop more effective prevention and treatment strategies, and evaluate program effectiveness; and infrastructure strengthening, investing in infrastructure and resources to support the program, including adequate healthcare facilities, equipment, and trained personnel (7, 8, 10).

## 2 Current status of vaccination programs

HPV vaccination programs have made remarkable strides in preventing HPV-related diseases, particularly cervical cancer (1, 11). Implementing widespread vaccination programs has led to notable reductions in the prevalence of diseases associated with human papillomavirus (HPV), including a decline in severe cervical abnormalities and genital warts (12, 13). Despite these positive outcomes, disparities exist in global vaccination coverage, with substantial differences between high-income countries (HICs) and low-income countries (LMICs). Low-resource settings often encounter challenges in vaccine accessibility, distribution infrastructure, and effective health education, hindering the equitable implementation of HPV vaccination programs.

In 2007, the United States, Canada, and Australia became the first countries to incorporate HPV vaccines into their national immunization programs (NIPs). As of April 2022, 120 countries had introduced prophylactic HPV vaccines into their NIPs. Despite this progress, only an estimated 13% of young girls globally are fully vaccinated, and HPV vaccines have yet to reach the populations most in need (14).

Within various vaccination programs, 47% predominantly targeted girls at the age of 12 years. However, LMICs typically targeted younger girls (9-10 years) compared to HICs, where the targeted age group was 11-13 years.

Most countries adopted a two-dose HPV vaccination schedule with a 6-month interval between doses. However, many countries reported using a 12-month interval between the first and second dose. In 2019, 76% of programs employed a single-cohort approach, where all girls in the targeted age group were vaccinated simultaneously. However, some programs transitioned from a multiple-cohort strategy, where girls were vaccinated in sequential age groups, to a single-cohort approach (15).

In certain LMICs where HPV vaccines are accessible, the immunization rates can vary markedly. Statistics indicate that 76.7% of individuals have received at least one dose of the HPV vaccine, 67.1% have received the second dose, and 31.1% have received the third dose (16, 17). Although there is a gradual increase in HPV vaccine coverage globally, in Mexico, the national vaccination program aimed at girls has shown a significant decrease in coverage during the COVID-19 pandemic. In 2021, HPV vaccine coverage among women in Mexico reached only 1%, a substantial decline attributed to the pandemic (15).

## 3 Human papillomavirus vaccines

Three HPV vaccines are licensed for use: bivalent (Cervarix), quadrivalent (Gardasil), and nonavalent (Gardasil 9). These vaccines prevent infection with HPV types 6/11/16/18/31/33/45/52/58 (18). All three vaccines are based on non-infectious recombinant type-specific L1 capsid proteins assembled into virus-like particles (VLPs) as immunogens (19). Humoral immunity against HPV is mediated by antibodies that recognize the L1 and L2 capsid proteins. B cells are activated by HPV antigens presented on MHC class II molecules by dendritic cells. A specific TH2-cell receptor recognizes the MHC-II/L1-L2 antigen complex. Activated B cells differentiate into memory and plasma B cells, producing HPV-specific antibodies (20).

Licensed HPV vaccines have undergone comprehensive safety, immunogenicity, and efficacy evaluations among young females aged 15-26. Consequently, organizations such as the World Health Organization (WHO) advocate the inclusion of the HPV vaccine in routine immunization schedules, with recommendations for girls starting as early as age 9. The vaccination regimen consists of a two-dose schedule for individuals receiving the initial dose before their 15th birthday, with an interval between doses ranging from 6 to 12 months. Alternatively, a three-dose schedule is recommended for individuals initiating vaccination at 15 or older and for immunocompromised individuals. This three-dose schedule involves administering the first dose and the second and third doses at 1-2 months and six months, respectively (21, 22).

In a post-hoc trial analysis, the effectiveness of the quadrivalent HPV vaccine was evaluated against cervical intraepithelial neoplasia (CIN) 2 or 3, adenocarcinoma *in situ* (AIS), and cancer. The analysis was conducted over seven years following vaccination, and the effectiveness of one dose was compared to two or three doses in preventing high-grade disease. The findings revealed that in a high-coverage setting, one dose of the vaccine demonstrated comparable effectiveness to two or three doses in preventing high-grade cervical lesions (23).

### 3.1 Efficacy

The human papillomavirus (HPV) vaccine has emerged as a crucial instrument in tackling the HPV-related health challenge. Recent data on the vaccine’s efficacy, immunogenicity, and safety parameters are extensively scrutinized to illuminate the underlying mechanisms contributing to its success.

Extensive studies have provided compelling evidence supporting the effectiveness of human papillomavirus (HPV) vaccines in preventing HPV infections and associated diseases. Drolet et al. conducted a comprehensive systematic review and meta-analysis, incorporating data from a substantial population of 60 million individuals. Their findings revealed that over a 5–8-year follow-up period, there was a remarkable decrease in the prevalence of HPV 16 and 18 by 83% (RR 0.17, 95% CI 0.11–0.25) among girls aged 13-19 years who received the vaccine, and by 66% (RR 0.34, 95% CI 0.23–0.49) among women aged 20-24 years. Similarly, in Australia, a study involving women aged 18-24 years demonstrated a substantial reduction in HPV 6/11/16/18 infections by 86% after completing the three-dose vaccination regimen and by 76% after receiving at least one dose, compared to unvaccinated counterparts. These findings underscore the profound impact of HPV vaccines in protecting individuals from HPV-related health concerns (12).

In countries exhibiting high uptake of the HPV vaccine, such as Australia and Denmark, substantial declines were observed in the prevalence and incidence of genital warts. The youngest age groups receiving vaccinations demonstrated the most significant reductions, with a 67% relative risk (RR) decrease (RR 0·33, 95% CI 0·24–0·46) among girls aged 15–19 years and a 54% RR decrease (RR 0·46, 95% CI 0.36–0.60) among women aged 20–24 years. Furthermore, the efficacy of the vaccination was highlighted by a 51% decrease in cervical intraepithelial neoplasia grade 2 or worse (CIN2+) after 5–9 years among screened girls aged 15–19 years (RR 0·49, 95% CI 0·42–0·58) and a 31% decrease among women aged 20–24 years (RR 0·69, 95% CI 0·57–0·84) (24). Similar positive outcomes were reported with the nonavalent HPV vaccine, which demonstrated an approximate 97% efficacy in preventing high-grade cervical, vulvar, and vaginal lesions due to additional HPV types (31, 33, 45, 52, 58). These findings strongly support the effectiveness of HPV vaccination in reducing the burden of HPV-associated diseases (25).

The HPV vaccine has proven effective in preventing HPV infection, genital warts, and high-grade cervical lesions (CIN2+ and CIN3+) (26). Additionally, it reduces the risk of invasive cervical cancer. A study by Lei et al. showed an 88% lower risk of cervical cancer among individuals vaccinated before 17 years of age (27). However, an Australian Vaccination Program report revealed that while cumulative HPV vaccination coverage increased from January 1, 2007, to December 31, 2019, the incidence rate of cervical cancer remained comparable between the post-vaccination and pre-vaccination periods. Notably, the mortality rate decreased by 12% (28).

HPV vaccination has a substantial impact on preventing HPV-related diseases, notably cervical cancer. Determining the vaccine’s long-term effectiveness in lowering the overall incidence of cervical cancer requires further investigation. Research also focuses on the vaccine’s efficacy based on the number of doses administered. Prelicensure vaccines demonstrated high efficacy with three-dose schedules. Studies investigating the noninferiority of two doses given at six or 12-month intervals revealed that ≥97.9% of individuals aged 9–14 years seroconverted to all nine HPV types, with a higher conversion rate observed in this age group when receiving two doses. Some studies have reported comparable effectiveness with three, two, and one doses (29), indicating the potential for fewer doses in the future.

### 3.2 Immunogenicity

Immunogenicity, a crucial factor influencing the effectiveness of vaccines, has been consistently demonstrated in HPV vaccines. These vaccines elicit robust and sustained immune responses, surpassing the immunity achieved through natural infection (30, 31). Seroconversion, defined as developing specific antibodies against the viral antigen, was observed in nearly 100% of individuals vaccinated with the 3-dose series. The titer of these antibodies increased after each dose and gradually declined over time following the completion of the vaccination course. Peak antibody titers induced by HPV vaccines are significantly higher than those generated after natural infection (32, 33).

Immunological memory elicited by HPV vaccines results in persistent antibody titers, providing long-term protection. Studies have demonstrated the stability of antibody levels over 9.4 years post-vaccination. This enduring immunity is attributed to the induction of memory B cells, which play a crucial role in maintaining immunological memory (34). Titers exhibit an inverse relationship with age, with higher titers observed in individuals aged 9-26 years compared to older age groups (24). Longitudinal evaluations of immune responses up to 24 months post-vaccination revealed a significant increase in geometric antibody titers (GMTs) for HPV type 16 (2.4-5.8-fold) and HPV type 18 (7.7-9.4-fold). These findings underscore the robust and sustained immunological response induced by HPV vaccines.

### 3.3 Cross-protection

Cross-protection against HPV types not targeted by the vaccines was investigated in both prelicensure clinical trials and post-licensure evaluations. Data obtained from randomized trials and observational studies focused on cross-protection among fully vaccinated groups revealed a statistically significant cross-protective efficacy with the bivalent vaccine, albeit with broad confidence intervals, against HPV types 6 and 11 at six- and twelve-months post-vaccination, with the most pronounced effect observed for HPV type 31. In the case of Gardasil, cross-protection was evident against HPV types 31 and 45, although preliminary data indicated that this cross-protection may be short-lived (35). Notably, the cross-protection antibody/immune response observed among participants who had received all three doses of the bivalent or quadrivalent vaccine is not directly comparable to the specific reactions elicited by the HPV vaccine types (35).

### 3.4 Vaccine safety

The safety of vaccines is of paramount importance in public health. Vaccines undergo rigorous safety evaluations to ensure their widespread use is justified. The HPV vaccine has been extensively studied, and its safety profile has been well-established. Large-scale clinical trials have demonstrated the safety of HPV vaccines. These trials have compared the safety profiles of HPV vaccines to those of other vaccines and found them comparable. Common adverse events associated with HPV vaccines are generally mild and include pain at the injection site and mild fever (36). The risk of serious adverse events is extremely low. After extensive analysis of the effectiveness, immunogenicity, and safety of HPV vaccination, the recommendations are highly compelling (**Table 1**).

**Table 1:** Types of prophylactic HPV vaccines in Mexico and recommendations through the years.

Year	Recommendations	HPV Vaccines
2009	Routine vaccination of a single birth cohort of girls aged 9–13 y with a 3-dose schedule	Bivalent Qudrivalent
2014	2-dose schedule, if starting series at age 9–14 y; 3-dose schedule for older girls/women or for immunocompromised persons	Bivalente Quadrivalent
2017	Vaccination of multiple cohorts of girls aged 9–14 y when vaccine first introduced; vaccination of other populations (females aged ≥15 y or males), only if feasible, affordable, cost-effective, and not diverting resources from primary target population or from cervical cancer screening programs	Bivalent Qudrivalent
2019	Owing to the global vaccine supply/demand imbalance, target girls aged 13 or 14 y, before they age out of the recommended primary target population, or schedule an extended interval of 3–5 y between the 2 doses, with dose 1 given to girls aged 9 or 10 y	Bivalent Qudrivalent
**2022**	A one or two-dose schedule for girls aged 9-14 years A one or two-dose schedule for girls and women aged 15-20 years Two doses with a 6-month interval for women older than 21 years	Bivalent Qudrivalent 9-valent HPV vaccine

## 4 Future directions

Preventive medicine is undergoing significant transformation due to the ongoing advancements in HPV vaccine technology. Researchers are actively optimizing vaccination schedules to ensure maximum efficacy and long-term protection. They are also exploring strategies to expand genotype coverage, aiming to develop vaccines that protect against a broader range of HPV strains. Furthermore, efforts are being made to address barriers to vaccine access, particularly in resource-limited settings, to ensure equitable distribution and utilization of these life-saving vaccines (37).

## 5 Vaccines in the secondary prevention

In secondary prevention strategies for CC, adjuvant HPV vaccination after treatment for CIN has been investigated. Several studies have evaluated the efficacy of this approach in reducing the risk of recurrence. Results consistently indicate a lower incidence of recurrent CIN 1, CIN 2+, and CIN3 in vaccinated groups compared to unvaccinated groups, with statistically significant p-values (p < 0.0001) (38).

Moreover, adjuvant HPV vaccination has demonstrated effectiveness in reducing the risk of anal intraepithelial neoplasia (p = 0.005) and recurrent respiratory papillomatosis. However, no significant differences were observed in recurrence rates for anogenital warts and vulvar intraepithelial neoplasia (39, 40). Further research is necessary to elucidate the precise role of HPV vaccination as an adjuvant therapy following primary treatment.

## 6 Conclusion

The review results provide compelling evidence of the significant impact of vaccination programs on HPV infection, anogenital warts, and high-grade cervical intraepithelial neoplasia, as well as a considerable proportion of other HPV-related cancers, such as oropharyngeal, vulva, vagina, and penis. Unfortunately, the coverage of vaccination programs in 2021 has been affected due to the COVID-19 pandemic, particularly in countries like Mexico. This situation has worsened the existing barriers and jeopardized the progress achieved in primary prevention. Therefore, it is crucial to address these challenges on programmatic, logistical, and financial fronts to continue the fight against HPV-related diseases.

To ensure that everyone has access to vaccines, support programs specifically tailored for marginalized populations must be created. Equitable access is critical in the case of HPV vaccines, as they play a crucial role in reducing health disparities. Improving strategies for increasing vaccination rates in underserved areas is essential to making it easier for girls to get vaccinated. Regarding increasing vaccination rates among girls in Mexico, balancing individual rights, public health priorities, and social justice considerations is necessary.

It’s recommended that the agencies involved coordinate their efforts to ensure fair and equal access to HPV vaccination. Any further delays in increasing coverage of the target population could lead to continued loss of life from preventable diseases and could also pose a significant financial burden on the health system.

## Author contributions

JG: Writing – original draft, Writing – review & editing. AC: Writing – original draft, Writing – review & editing. VM: Writing – original draft, Writing – review & editing.
